# Muslim Women’s use of contraception in the United States

**DOI:** 10.1186/s12978-017-0439-6

**Published:** 2018-01-05

**Authors:** Henna Budhwani, Jami Anderson, Kristine R. Hearld

**Affiliations:** 1 Department of Health Care Organization and Policy, School of Public Health, University of Alabama at Birmingham (UAB), 310D Ryals Public Health Building, 1665 University Boulevard, Birmingham, AL 35294 UK; 2 Health Services Administration, University of Alabama at Birmingham, Birmingham, UK

**Keywords:** Women’s health, Islam, Contraception, Reproductive health

## Abstract

**Background:**

American Muslim women are an understudied population; thus, significant knowledge gaps exist related to their most basic health behaviors and indicators. Considering this, we examined American Muslim women’s contraception utilization patterns.

**Methods:**

Self-reported data collected in late 2015 were analyzed. Women who identified as Muslim, were at least 18 years old, sexually active, and current residents of the United States (*n* = 224) met the inclusion criteria. Convenience sampling was employed. Multivariate logistic regression models estimated associations between demographics, marital status, ethnicity, nativity, health insurance, religious practice, and contraception use.

**Results:**

Identifying as Muslim, in general, was significantly associated with greater odds of using contraception in general and condoms compared to American Muslim women who identify as Sunni. Identifying as Shia was associated with greater odds of using oral contraceptive pills relative to Sunni respondents. South Asian ethnicity was associated with higher odds of using oral contraceptive pills compared to those of Middle Eastern or North African ethnicity.

**Conclusions:**

Findings suggest American Muslim women’s contraception utilization patterns share certain similarities with both American women in general and disadvantaged racial and ethnic minority groups in the United States, implying that factors that influence American Muslim women’s use of contraceptives are possibly countervailing and likely multifaceted. More research is needed to accurately identify associates of contraceptive use in this population. This work serves as a starting point for researchers and practitioners seeking to better understand reproductive health decision in this understudied population.

## Plain English summary

There is little scientific data available on the health-related knowledge, behaviors, and attitudes of American Muslim women. This knowledge gap is not surprising, considering American Muslim women are part of a religious minority population that experiences ongoing stigma; some American Muslim women are immigrants, and some are racial and ethnic minorities. To address this knowledge gap, we collected self-reported survey data from American Muslim women across the United States. We then statistically analyzed contraception utilization patterns of our respondents. Our study design and the variables included were informed by previous research on minority populations in the United States and social theory. We found that being a Shia Muslim or Muslim, in general (no sect declared) was significantly associated with higher odds of using contraception. Respondents identifying as South Asian had high odds of using oral contraceptive pills compared to Middle Eastern and North African respondents. Our findings suggest these American Muslim women’s contraception utilization patterns share similarities with American women in general and with disadvantaged minority groups. More research is needed to better understand barriers and facilitators of contraceptive use in this population.

## Background

Muslim women in the United States are an understudied population; thus, significant knowledge gaps persist pertaining to their most basic health indicators and behaviors [1, 2]. This is, in part, because religious identification is not routinely collected in social science studies, making it impossible to examine outcomes across religion. In the studies that collect this information, often in large nationally representative datasets, religious identification is restricted and difficult (if not impossible) to access. Therefore, researchers and clinicians know little about American Muslim women’s contraceptive utilization; whereas in comparison, studies on contraception in immigrant, racial, and ethnic minority groups are abundant [3–7]. These studies have found 62–75% of American women aged 15–44 used some form of contraception with oral contraceptive pills being the most popular, followed by female sterilization, and male condoms [8–11]. In contrast, the United Nations reported utilization of any contraception was 38.5% in Pakistan, 59.7% in Egypt, 38.6% in Saudi Arabia, 67.8% in Morocco, 62.4% in Lebanon, 63.8% in Tunisia, and 62.3% in Indonesia, all Muslim majority countries [12], illustrating significant variability in contraceptive use across nations. The range of these rates suggests contraceptive utilization is associated with a spectrum of factors and cannot simply be extrapolated from one group to another.Since Muslim women residing in the United States have intersectionality in their cultural profile -- as religious, racial, and ethnic minorities, possibly identifying as an immigrant, while also being American -- their health outcomes and health behaviors are likely nuanced not aligning completely with American women, in general, Muslim women living abroad, or racial and ethnic minority women in the United States. Considering these knowledge gaps and the multifaceted cultural profile of American Muslim women, we conducted an exploratory study to 1) identify demographic and sociocultural (including religious) associates of contraceptive utilization, and 2) to detect if these women’s contraceptive use patterns align more with those of American women, in general or with heavily foreign-born minority populations. To inform the design of this study, we explored prior contraception research on minority and non-minority populations to identify potentially pertinent factors to include in the analyses, as well as applicable theories.

Not only does contraceptive utilization vary across nations, it fluctuates widely across racial and ethnic groups in the United States [13–16]. American white (single race) women reported using highly effective forms of contraceptives, specifically oral contraceptive pills and condoms, more often than African American and Hispanic women [10, 11, 13, 14]. Racial and ethnic minorities were also less likely to use contraception all together and tended to use lower-efficacy methods more frequently than white, America women [10, 11, 13, 14]. Consequently, racial and ethnic minorities in the United States were twice as likely to experience an unintended pregnancy compared to white women [15]. Contraceptive methods that were in the direct control of the woman were found to be more popular among ethnic minorities and were used when the woman desired to maintain her personal control over reproduction or to obfuscate contraception utilization from her partner [14, 16]. A study in California found that African American and Hispanic women were more likely to use injectable contraceptives and emergency contraceptive pills compared to oral contraceptive pills, potentially to minimize risk that her partner would become aware of her contraception use [16]. Building upon findings related to contraceptive utilization, Gomez and Marin identified three primary factors influencing the method of contraception selected: the role of contraception in preventing pregnancy and/or disease, the influence of cultural sexual gender norms, and complexities of contraception with a steady partner [17]. These three factors recognize the influence of gender-based power differentials and highlight the impact that a power imbalance has on contraceptive choices, which is particularly relevant to socially conservative, patriarchal sub-populations such as American Muslims [17].

Other theorists have examined factors which influence contraceptive use, and their theoretical frameworks suggest there are three behavioral factors that significantly influence the use of and type of contraceptive method selected: 1) Autonomous decision making authority of the woman, 2) Ability to negotiate with her health care provider (and health insurance coverage) about contraceptive preferences, and 3) Influence of cultural subjective norms directly and indirectly related to contraception [18–23]. With respect to condom use, the theory of planned behavior suggests that an individual’s decision to use or not to use condoms reflects a combination of beliefs about the benefits of use and the barriers to use [21]. Although informative to understanding motivations to contraception use, these theories fail to incorporate the influence of stigma. Stigma is the process by which a group is labeled as socially deviant and devalued due to attributes or behaviors deemed as discrediting, such as American Muslim women who use contraception even though their religious or cultural orientation prohibits pre-marital sex and suggests the purpose of intercourse is for procreation; these women could become stigmatized by others who do not use contraception or may internalize stigma even without being confronted about the behavior [24–29].

Nativity may also play a role in the decision to use contraceptives. The healthy migrant effect asserts that foreign-born individuals are healthier and more resilient than their American born peers; this is due to a selection bias in that only the healthiest individuals from a given population immigrate to the United States [30–32]. This protective effect is magnified in higher-income and highly educated immigrants, indicating socioeconomic status, is a powerful associate of health behaviors in foreign-born populations [30–32]. Although the healthy migrant effect has been historically applied to physical health outcomes, recent studies have found this effect to be pertinent to mental health outcomes, as well [30–32]. Since many American Muslim women were born abroad, their nativity may be associated with utilization or non-utilization of contraceptives. In addition, immigrants often hail from cultures that avoid health care associated with sexual engagement (stigmatized), perceived premature sexual debut, and gendered norms and expectations [33–35]; thus, nativity may be associated with contraceptive utilization in American Muslim women. Considering the countervailing forces affecting American Muslim women’s potential health behaviors and significant gaps in knowledge related to this population, we believe examining contraceptive utilization patterns in American Muslim women is a worthy endeavor and will provide valuable insights into the family planning choices of this infrequently studied population.

## Methods

### Study design and participants

The Muslim Women’s Health project was conducted for three months between September 2015 and December 2015. The goal of this study was to collect self-reported, exploratory data on a range of health behaviors and outcomes from American Muslim women. The primary outcomes for this particular sub-study related to contraceptive use; defined below. Respondents were recruited through online social networks, email requests, and postings made to online Muslim communities. To complete the survey or to learn more about this study. Information on the Muslim Women’s Health project, the funding institution (University of Alabama at Birmingham School of Public Health), principal investigator, and ethical approval were available at this portal. Respondents were not compensated for their participation.

Women who self-identified as Muslim, were at least eighteen years old, and were current residents of the United States were eligible to participate. Every respondent was asked to answer the same question in the same sequence -- question delivery was not randomized; respondents were able to skip any question, after the eligibility screening. Average time to complete the survey was about fifteen minutes due to the inclusion of a number of skip patterns allowing respondents to skip full sections that were not applicable. For example, if a respondent answered that she had never used any form of contraception; she was not asked questions about types of contraceptive used. Although online surveys have limitations, such as sampling bias, one major benefit is the ability to engage difficult-to-reach populations, including stigmatized populations, minority enclaves, and groups fearing persecution [36]. Due to the employment of convenience sampling without unique personal identifiers, a response rate could not be calculated. The base sample size was *N* = 373. Our analysis of contraceptive utilization was limited to respondents who reported being sexually active (*N* = 224).

### Outcome measures

Our primary outcomes were use of contraception (any method), oral contraceptive pills, condoms, and the withdrawal method. Respondents were first asked if they had used contraception and then the type of contraceptive method was assessed by a 10-item multi-response question inquiring about the type of contraception used including: withdrawal method, condoms, oral contraceptive pills, intrauterine device, sponge, female condom, vaginal ring, diaphragm, contraceptive patch, and subdermal implant. Respondents who answered “yes” to contraceptive use were coded as 1 while respondents who answered “no” were coded as 0. Similarly, use of oral contraceptive pills, condoms, and the withdrawal method were assessed with binary variables. Respondents who answered “yes” were coded as a 1 and responses of “no” were coded as 0.

### Independent measures

To examine the relationship between individual characteristics and contraception use, we included independent measures of personal demographics, socioeconomic status, health insurance, and religion. Demographic characteristics were assessed four variables: age, marital status, ethnicity, and nativity. Age was operationalized with a continuous variable while marital status was assessed by a binary variable: married or not married. Ethnicity was operationalized with a series of binary indicators including Middle Eastern or North African, South Asian, and other (referent). Nativity was assessed with a single dummy variable, coded as 1 if the respondent was born in the United States and 0 if the respondent was born elsewhere. Socioeconomic status was assessed with two variables: annual household income grouped into six categories: under $24,999 (referent), $25,000–74,999, $75,000–99,999, and $100,000 and over, and education. Education was coded into four categories: high school education (referent), some college or vocational school, college graduate, and graduate or professional school. Health insurance was assessed with three categories: no insurance (referent), private insurance, and public insurance. Religion included two variables of religious sect and worship attendance. Religious sect was assessed with three categories: general Islam (does not identify as Shia or Sunni, referent), Shia, and Sunni. Worship attendance included five categories: less than once a month (referent), once a month, multiple times a month, once a week, and multiple times a week.

### Statistical analysis

Frequencies described sexually active American Muslim women’s contraceptive use, demographics, socioeconomic status, religion, and health insurance status. Logistic regressions were used to estimate the relationship between demographics, socioeconomic status, religion, and the use and type of contraception. All analyses were conducted using Stata version 13.0.

### Ethics, consent and permissions

Ethical approval was obtained from University of Alabama at Birmingham’s Institutional Review Board (IRB, #X150413001). Informed consent was collected via an IRB approved online form.

## Results

Table 1 provides sample characteristics (*N* = 224). The majority of respondents reported using contraceptives (79.5%). Approximately 66% used oral contraceptive pills (65.6%), 66.1% used condoms, and 32.1% used the withdrawal method. Average age was 32.2 years and ranged from 18 to 49 years. Two thirds of the respondents were married (66.5%). With respect to ethnicity, 44.2% of the respondents identifying as having South Asian ethnicity, 25.9% identified as having Middle Eastern or North African ethnicity, 29.9% identifying as having an ethnicity other than South Asian or Middle Eastern/North African. About 38% of the respondents were born in the United States (38.4%), 53.9% attended graduate or professional school, and 43.3% reported an annual household income of over $100,000. Most respondents carried private insurance (83.1%); only 5.6% were uninsured. About 38% (38.4%) identified as Sunni, compared to Shia (46.4%), and 15.2% identified as Muslim, in general (neither Shia nor Sunni). About 42% of the sample attended worship once a week or more frequently (42.34%).

**Table 1:** Characteristics of Muslim Women

	Muslim Women *N* = 224 (%)
*Contraception*	
Contraception use	79.46
Oral contraceptive pill use	65.63
Condom use	66.07
Withdrawal use	32.14
IUD use	15.63
*Demographic Characteristics*	
Age (M/SD)	32.19/0.50
Marital Status	
Married	66.52
Single/Widowed/Divorced	33.48
Ethnicity	
Middle Eastern or North African	25.89
South Asian	44.20
Other Ethnicity	29.91
Nativity	
U.S. Born	38.39
Education	
High School	2.74
Some College or Vocational	11.42
College Graduate	31.96
Graduate or Professional School	53.88
*Socioeconomic Characteristics*	
Household Income	
Under $24,999	9.77
$25,000 to $74,999	34.88
$75,000 to $99,999	12.09
Over $100,000	43.26
Employment Status	
Employed	73.21
Not Employed	26.79
*Religious Characteristics*	
General Islam	15.18
Shia	46.43
Sunni	38.39
Worship attendance	
Less than once a month	38.75
Once a month	4.05
Multiple times a month	14.86
Once a week	17.57
Multiple times a week	24.77
*Health Insurance Coverage*	
Uninsured	5.61
Public Insurance	11.21
Private Insurance	83.18

Table 2 presents the odds ratios and confidence intervals from the logistic regression analysis of contraception use and individual characteristics. Married respondents were associated with 2.7 greater odds of contraception use, relative to not married respondents (OR:2.663, CI:1.073, 6.609). With respect to ethnicity, Muslim women identifying as having South Asian ethnicity were associated with 3.1 greater odds of using oral contraceptive pills relative to respondents identifying as having Middle Eastern or North African ethnicity (OR:3.071, CI:1.154, 8.176). Respondents identifying as having ethnicities other than South Asian or Middle Eastern/North African were associated with 4.0 greater odds of using oral contraceptive pills, relative to respondents identifying as having Middle Eastern or North African ethnicity (OR:4.046, CI:1.538, 10.642). Respondents carrying private insurance were associated with lower odds of using the withdrawal method relative to respondents without insurance (OR:0.196, CI:0.044, 0.867).

**Table 2:** Multivariate regression results, simultaneous entry (*N* = 224)

	Birth Control	Oral Contraceptive Pills	Condom	Withdrawal
	Odds Ratio, 95% CI	Odds Ratio, 95% CI	Odds Ratio, 95% CI	Odds Ratio, 95% CI
*Demographic Characteristics*				
Age				
18–29	referent	referent	referent	referent
30 to 39	0.560 (0.216, 1.452)	0.600 (0.266, 1.354)	0.607 (0.268, 1.375)	1.129 (0.499, 2.554)
40 to 49	1.210 (0.308, 4.757)	0.516 (0.181, 1.475)	0.909 (0.307, 2.695)	1.724 (0.611, 4.864)
Marital Status				
Not Married	referent	referent	referent	referent
Married	2.663 (1.073, 6.609)*	1.737 (0.786, 3.834)	1.599 (0.731, 3.497)	0.596 (0.267, 1.331)
Ethnicity				
Middle Eastern or North African	referent	referent	referent	referent
South Asian	1.728 (0.576, 5.184)	3.071 (1.154, 8.176)*	1.334 (0.508, 3.503)	0.849 (0.299, 2.410)
Other Ethnicity	2.475 (0.811, 7.552)	4.046 (1.538, 10.642)**	2.271 (0.880, 5.859)	0.795 (0.285, 2.214)
Nativity				
Foreign Born	referent	referent	referent	referent
U.S. Born	0.748 (0.039, 7.952)	0.878 (0.421, 1.834)	0.856 (0.408, 1.799)	2.130 (0.996, 4.554)
*Socioeconomic Characteristics*				
Education				
High School	referent	referent	referent	referent
Some College or Vocational	0.560 (0.039, 7.952)	2.617 (0.227, 30.142)	0.495 (0.236, 1.042)	0.689 (0.028, 17.164)
College Graduate	0.955 (0.074, 12.354)	3.801 (0.359, 40.207)	0.260 (0.086, 0.789)	2.288 (0.099, 52.583)
Graduate/Professional	1.547 (0.117, 20.466)	4.475 (0.416, 48.141)	0.812 (0.066, 9.974)	3.036 (0.129, 71.400)
Household Income				
Under $24,999	referent	referent	referent	referent
$25,000 to $74,999	1.035 (0.197, 5.446)	0.309 (0.069, 1.381)	1.072 (0.288, 3.993)	1.475 (0.294, 7.405)
$75,000 to $99,999	1.375 (0.200, 9.429)	0.342 (0.060, 1.938)	1.553 (0.316, 7.656)	3.312 (0.552, 19.858)
Over $100,000	1.583 (0.271, 9.261)	0.388 (0.079, 1.918)	1.737 (0.422, 7.146)	5.310 (0.991, 28.465)
*Religion Characteristics*				
Islam				
Sunni	referent	referent	referent	referent
Shia	2.769 (1.056, 7.262)*	1.275 (0.552, 2.945)	2.032 (0.886, 4.661)	0.870 (0.361, 2.010)
General Islam	5.491 (1.073, 28.101)*	0.868 (0.306, 2.466)	3.667 (1.136, 11.832)*	1.834 (0.636, 5.289)
Worship Attendance				
Less than once a month	referent	referent	referent	referent
Once a month	1.577 (0.149, 16.712)	3.808 (0.391, 37.129)	1.494 (0.244, 9.151)	5.954 (1.098, 32.280)*
Multiple times a month	0.532 (0.149, 1.903)	1.583 (0.484, 5.178)	0.655 (0.223, 1.928)	1.424 (0.506, 4.006)
Once a week	0.373 (0.122, 1.142)	0.724 (0.275, 1.906)	0.556 (0.208, 1.485)	0.622 (0.211, 1.834)
Multiple times a week	0.462 (0.153, 1.395)	0.383 (0.151, 0.973)*	0.485 (0.187, 1.257)	1.265 (0.474, 3.376)
*Health Insurance Coverage*				
Uninsured	referent	referent	referent	referent
Public Insurance	6.998 (0.886, 55.294)	1.157 (0.203, 6.587)	1.014 (0.183, 5.628)	0.150 (0.021, 1.088)
Private Insurance	1.802 (0.428, 7.589)	1.737 (0.424, 7.113)	0.996 (0.248, 3.993)	0.196 (0.044, 0.867)*

*p < 0.05; **p < 0.01; ***p < 0.001

As to religious characteristics, respondents identifying as Shia were associated with 2.8 greater odds of contraceptive use, relative to respondents identifying as Sunni (OR: 2.769, CI:1.056, 7.262). Respondents identifying as Muslim, in general, were associated with 5.5 greater odds of using any form of contraception (OR:5.491, CI:1.073, 28.101) and 3.7 greater odds of using a condom (OR: 3.667, CI:1.136, 11.832) compared to respondents identifying as Sunni. Worship attendance of multiple times a week was associated with lower odds of use of oral contraceptive pills, compared to respondents who report worship attendance of less than once a month (OR:0.383, CI:0.151, 0.973). Respondents who reported worship attendance of once a month were associated with 6.0 higher odds of withdrawal than respondents who reported worship attendance of less than once a month (OR:5.954, CI:1.098, 32.280).

Certain non-statistically significant, but directionally relevant trends emerged, as well. Nativity did not have a statistically significant effect; however, American-born Muslim women had lower odds of using any contraception, oral contraceptive pills, and condoms. This reversed with utilization of the withdrawal method; with American-born women having higher odds to using the withdrawal method compared to their foreign-born peers. Education, although also not significant, did -- for the most part -- function as anticipated. Respondents with a graduate level of education utilized any contraception, oral contraceptive pills, and the withdrawal method at higher odds than respondents with a high school or lower level of educational attainment. Condom use was an outlier, with all higher levels of education having lower odds of condom use compared to the high-school referent category, possibly suggesting a bias against condom use in this particular population.

## Discussion

Compared to the general public, racial and ethnic minorities have repeatedly been shown to have lower rates of contraceptive utilization and frequently use lower-efficacy methods than non-minority women [3–11, 13, 14]. About 79% of the American Muslim women in our study reported any contraceptive use, which is higher than the 65–72% found in American women, in general. The majority of our sample also maintained private insurance, and 43% reported an annual household income over $100,000. By these measures, it is not surprising to find such high rates of contraceptive use considering both household income and health care access are associated with preventative health care utilization in other racial and ethnic minority groups.

In contrast to the high rates of contraceptive utilization, we found a limited range of methods used; we found usage of oral contraceptive pills, condoms, and the withdrawal method. We found minimal utilization of the contraceptive patch, injectable contraception, and implants (statistics not reported). This gap between contraceptive methods employed in minority and non-minority women is growing and may allude to the impact of social and community circumstances that, with respect to American Muslim women, are unique to their culture and beliefs pertaining to reproduction [37, 38]. Thus, in some ways our sample’s behaviors aligned with American women generally (e.g., relatively high rates of contraceptive utilization), while in others, their behaviors reflected what would be expected from a heavily foreign-born, racial and ethnic minority population (e.g., limited types of contraceptives utilized).

Religion and Islamic sect may influence the acceptability, perceptions, and attitudes of contraceptive use and may strongly influence the individual decision-making process. About 38% of our sample identified as Sunni Muslims. Shia respondents and respondents who identified as ‘general Islam’ -- a category we suspect may include those who are religiously more secular but culturally still identify as Muslim -- had higher odds of using contraception relative to Sunni Muslims. Considering Islam as a social modality, precise cultural criteria may define the reproductive practices within these societies and heavily influence the contraceptive choices acceptable to American Muslim women [39–41]. Many Muslim societies discourage use of any contraception, and social networks actively inhibit the use of contraceptives through social pressure (and stigma); therefore, it is noteworthy that we found statistical differences in contraceptive utilization between Islamic sects [41]. Additionally, although not statistically significant, a notable trend was found in which respondents who attended worship more often had lower odds of utilizing contraceptives. While a discussion on Islamic practices and contraceptive use is outside the scope of this study, it is important to consider that some American Muslim women may have emigrated from areas where reproductive practices are socially controlled by religious ideology but now reside in a nation wherein these pressures are less prevalent.

Limitations should be considered when applying these findings. Although our sample size is larger than other studies on Muslim health, it is still small compared to the population of Muslim women residing in the United States. Selection bias existed; respondents were more educated and wealthier than Americans in general, and we suspect even in comparison to the wider American Muslim population. Yet, it is noteworthy that these high income, highly educated American Muslim women had contraceptive utilization rates that outpaced contraceptive use found in the highest quintile of Muslim women in nations from which respondents migrated, such as Pakistan [42]. Although online surveys have benefits, including their ability to reach stigmatized populations, there are limitations of online surveys including the likelihood of not reaching the most disenfranchised including those who do not speak or read English and those with limited technology access or proficiency.

## Conclusions

Given the complexities of delivering reproductive health care and increasing access to contraceptives within secular societies, it is likely that additional confounding arises when considering the cultural preferences of Muslim Americans [43]. Prior studies suggest that barriers to effective contraceptive counseling for American Muslim women include issues of modesty, gender preference in healthcare providers, and misconceptions regarding illness causation [44, 45]; however, we found a high rate of contraceptive utilization in this well-educated and wealthy sample suggesting that cultural preferences may be overcome by improved socioeconomic status specifically related to contraceptive use. Our findings contribute to new scientific knowledge that will enable public health practitioners, clinicians, and researchers to better understand American Muslim women, this population’s heterogeneity, and variability of health and sociocultural behaviors relating to their contraception utilization.

## Abbreviations

CI: Confidence Interval
OR: Odds Ratio
